# Characterization of vaginal microbiota across female reproductive phases

**DOI:** 10.3389/fmicb.2025.1599965

**Published:** 2025-07-04

**Authors:** Qingzhi Zhai, Yuan Gao, Mingyang Wang, Li Li, Lian Li, Jianghua Li, Ying Ma, Ke Niu, Mingxia Ye

**Affiliations:** ^1^Department of Obstetrics and Gynecology, The Seventh Medical Center of PLA General Hospital, Beijing, China; ^2^The Fourth Medical Center of PLA General Hospital, Beijing, China

**Keywords:** vaginal microbiota, reproductive phase, 16S rRNA sequencing, hormonal variations, microbial diversity

## Abstract

**Background:**

The vaginal microbiota plays a crucial role in women’s reproductive health, but its composition and function throughout different female reproductive phases remain poorly understood. This study aimed to investigate the dynamic variations in vaginal microbial communities corresponding to different hormonal states throughout the female reproductive phase.

**Methods:**

Vaginal samples were collected from 150 healthy women across five reproductive phases: follicular phase, luteal phase, early pregnancy, lactation, and menopause (30 samples per group). 16S rRNA sequencing was used to analyze the microbial composition. Alpha and beta diversity analyses were performed, and random forest models were constructed to identify key microbial taxa associated with each phase. Functional profiling was conducted using Clusters of Orthologous Groups of Proteins (COG) and Kyoto Encyclopedia of Genes and Genomes (KEGG) databases.

**Results:**

Microbial communities show significant variations across the different reproductive phases. *Firmicutes* dominate in the early reproductive phases but decrease notably during lactation and menopause. Conversely, *Bacteroidetes*, *Actinobacteria*, and *Proteobacteria* increase in later phases. At the genus level, *Lactobacillus* maintains a higher abundance during reproductive phases but declines during lactation and menopause. Peak microbial diversity occurs during lactation. Functional predictions reveal distinct phase-specific microbial functions. The follicular phase is enriched in pathways associated with nitrotoluene degradation and flavonoid biosynthesis, potentially reflecting estrogen-mediated regulation. Lactation shows enrichment in pathways related to protein digestion and fatty acid metabolism, consistent with increased nutritional demands. Menopause is characterized by enrichment in steroid hormone biosynthesis pathways. In early pregnancy, enrichment of functions related to the adenine nucleotide translocator and phosphate carrier reversible complex suggests functional adaptation of the microbiota to early gestational physiology.

**Conclusion:**

Our study reveals dynamic variations in vaginal microbiota across reproductive phases, closely linked to hormonal states, and highlights potential microbial targets for enhancing women’s reproductive health.

## 1 Introduction

Microbial communities exhibit intricate interactions with host physiology and metabolism, exerting a significant influence on essential functions including nutrient assimilation, immune system maturation, and pathogen defense (Kaur et al., 2020; Wang et al., 2020; Grice and Segre, 2012). Within the female reproductive tract, the vaginal microbiota constitutes a critical ecosystem, predominantly composed of diverse *Lactobacillus* species and other anaerobic bacteria such as *Gardnerella* and *Atopobium*, forming a complex symbiotic network (Punzón-Jiménez and Labarta, 2021; Alonzo Martínez et al., 2021; Redelinghuys et al., 2017). These microorganisms play a pivotal role in maintaining vaginal homeostasis, particularly through the modulation of pH and the suppression of pathogenic microbial proliferation. Additionally, these bacteria synthesize hydrogen peroxide and bacteriocins, further augmenting the antimicrobial properties of the vaginal milieu (Jack et al., 1995; Strus et al., 2006). The ecological dynamics of the vaginal microbiota significantly contribute to reproductive health by establishing a protective barrier against potential pathogens and maintaining the physiological equilibrium of the vaginal environment.

Typically, the composition and stability of the vaginal microbiota are influenced by various factors, including individual dietary habits, behavioral patterns, hygiene practices, age, genetic predispositions, and fluctuations in sex hormone levels throughout the female reproductive phase and menopause (Cagnacci et al., 2020; Graham et al., 2021). Previous studies indicate that sex hormones (e.g., estrogen, progesterone, and testosterone) directly stimulate the growth and colonization of lactobacilli while also indirectly regulating the dynamics of the vaginal microbiota (Graham et al., 2021; Yoon and Kim, 2021; Kunc et al., 2015; Maffei et al., 2022). Significant hormonal fluctuations during different reproductive phases and menopause profoundly alter the composition and functionality of the vaginal microbiota. Differences in female sex hormone levels are associated with various systemic and reproductive diseases (Yoon and Kim, 2021; Taneja, 2018; Wang et al., 2019; Li et al., 2018). Furthermore, sex hormone levels influence innate and adaptive immune responses that safeguard the female reproductive tract against pathogen invasion (Kaur et al., 2020; Wira et al., 2011). Thus, these physiological factors collectively influence the dynamic variations in composition and function of the vaginal microbiota throughout a woman’s lifespan.

Women experience various physiological phases throughout their reproductive phase: follicular phase, luteal phase, early pregnancy, lactation, and menopause, each characterized by significant differences in sex hormone levels. The follicular phase initiates the menstrual cycle with rising estrogen levels, preparing the uterine lining for potential embryo implantation. Following ovulation, the luteal phase features increased progesterone levels that stabilize the uterine lining for possible pregnancy. During pregnancy, *lactobacilli*, especially *L. crispatus* and *L. iners*, dominate the vaginal microbiota, supporting embryo development with low bacterial diversity. After childbirth, there’s a notable shift in vaginal microbiota, marked by reduced lactobacilli and increased diversity of bacteria like *Peptoniphilus*, *Anaerococcus*, *Prevotella*, *Gardnerella*, *Atopobium*, and *Streptococcus*, often leading to higher diversity (Wei et al., 2021; Grobeisen-Duque et al., 2023; Pépin et al., 2011). Studies indicate that lactobacilli-dominated communities resembling CST-III and CST-IV, as defined by Ravel et al., are common postpartum, with *L. iners* frequently increasing over time (Ravel et al., 2011; Marconi et al., 2020; Gerson et al., 2021; Novak et al., 2023). Unlike more stable *lactobacilli* communities, those dominated by *L. iners* tend to be less stable and more diverse, often transitioning to CST-IV profiles. During lactation, elevated prolactin levels alongside lower estrogen and progesterone affect vaginal microbiota, with estrogen directly promoting *lactobacilli* growth and colonization by enhancing their proliferation and lactic acid secretion through receptor binding (Deka et al., 2021; Hallmaier-Wacker et al., 2019). Sex hormones also indirectly influence microbial communities by affecting vaginal epithelial cell secretions, including increased glycogen synthesis that nourishes *lactobacilli* growth (Bradley et al., 2018). During menopause, declining estrogen levels reduce dominant *Lactobacillus* populations, raising vaginal pH and increasing microbial diversity, potentially elevating infection risk and impacting urogenital health (Cagnacci et al., 2020; Muhleisen and Herbst-Kralovetz, 2016).

While studies have demonstrated significant impacts of sex hormones on vaginal microbiota, the dynamic relationship between hormone fluctuations across various physiological states and vaginal microbiota remains unclear. Understanding this link is crucial for preventing and treating reproductive tract infections, and for maintaining vaginal health through personalized treatment strategies. Current research has mainly focused on how sex hormones affect vaginal microbiota during specific physiological phases, but comprehensive studies covering changes throughout the entire reproductive phase and menopausal phases are lacking. Such research could offer a comprehensive overview of community dynamics in the vaginal microenvironment and how they evolve over a woman’s lifetime.

The present study, approved by the Ethics Committee of the General Hospital of the People’s Liberation Army, recruited 150 healthy female participants who attended outpatient services between February and December 2024, encompassing various physiological phases: follicular phase, luteal phase, menopause, early pregnancy, and lactation. Utilizing the GY66 vaginal microecology detection platform, a Polymerase Chain Reaction (PCR)-based assay, we excluded common vaginal infections such as bacterial vaginosis, vulvovaginal candidiasis, and trichomoniasis, and ensured participants had a normal body mass index. Employing 16S rRNA gene sequencing, we systematically examined the dynamic variations in vaginal microbiota across distinct reproductive phases. Additionally, we developed a model based on microbial community abundances exhibiting significant dynamic variations to predict sex hormone status. The findings from this research offer valuable insights into understanding the nature of vaginal microbiome imbalances, potentially informing the development of enhanced strategies for women’s gynecological and reproductive health care and management.

## 2 Materials and methods

### 2.1 Participant enrollment and classification criteria for reproductive phase study

With the approval of the Ethics Committee of the PLA General Hospital, subjects provided informed consent and were enrolled from the healthy outpatient population at our hospital between February and December 2024. Participants were categorized based on their menstrual cycle into the following phases: follicular (Group 1; age range: 25–44 years old), luteal (Group 2; age range: 29–43 years old), early pregnancy (Group 3; age range: 26–39 years old), lactation (Group 4; age range: 25–38), and menopausal (Group 5; age range: 47–73). Each group included 30 subjects, resulting in a total of 150 participants (Supplementary Table S1).

Additionally, the follicular and luteal phases were defined through menstrual cycle tracking and gonadal hormone assessments. The menopausal phase was defined by 1 year of amenorrhea, with gonadal hormone tests indicating ovarian function decline, while excluding endocrine disorders such as premature ovarian failure. The early pregnancy phase included women within 12 weeks of amenorrhea, confirmed by blood HCG levels and ultrasound indicating intrauterine pregnancy. The lactation period was defined as 42 ± 3 days postpartum, with participants actively breastfeeding and without resumption of menstruation.

### 2.2 Eligibility criteria for participants

The inclusion criteria for this study are as follows: participants must be healthy women with a clearly defined physiological menstrual cycle, as determined by menstrual history or gonadal hormone assays. They must have a normal body mass index (BMI) ranging from 18.5 to 24, and be free from bacterial vaginosis, vulvovaginal candidiasis, trichomoniasis, aerobic vaginitis, and mixed vaginitis based on vaginal secretion analyses. Additionally, participants must not have engaged in sexual activity or used intravaginal medications within the past 3 days (as confirmed by structured questionnaires) and should not have undergone any oral or intravenous anti-inflammatory treatment within the past week.

The exclusion criteria include individuals with HPV infection confirmed by cervical screening, those with vulvar pruritus without confirmed microbial infection upon vaginal secretion analysis, and those who have used povidone-iodine for vaginal cleansing or lubrication prior to sampling. Furthermore, women taking oral gonadal hormones to regulate their menstrual cycle and individuals with chronic conditions such as hypertension or diabetes that necessitate long-term oral medication are excluded from participation.

### 2.3 16S RNA sequencing

#### 2.3.1 DNA extraction and quality control

Genomic Deoxyribonucleic Acid (DNA) was extracted from samples using the Cetyltrimethylammonium Bromide (CTAB) or Sodium Dodecyl Sulfate (SDS) method. DNA concentration was measured by Nanodrop, while purity and integrity were assessed via 1% agarose gel electrophoresis.

#### 2.3.2 Amplicon generation and PCR

DNA was diluted to 1 ng/μL. 16S rRNA genes were amplified using specific barcoded primers. PCR reactions (30 μL) contained 15 μL High-Fidelity PCR Master Mix, 0.2 μM of each primer, and ~10 ng template DNA. Thermocycling conditions: 98°C for 1 min; 30 cycles of 98°C for 10 s, 50°C for 30s, 72°C for 30s; final extension at 72°C for 5 min.

#### 2.3.3 PCR product analysis and purification

Products were visualized on 2% agarose gel with SYBR Green. Samples showing bright bands between 400 and 450 bp were selected. Amplicons were pooled equally and purified using the TIANgel Purification Kit.

#### 2.3.4 Library preparation and sequencing

Libraries were prepared using TIANSeq Fast DNA Library Prep Kit. Quality was assessed using Qubit@ 2.0 Fluorometer and Agilent Bioanalyzer 2100. Sequencing was performed on the Illumina platform using 2 × 250 bp paired-end protocol.

### 2.4 Sequencing data preprocessing and taxonomy assignment

Microbiome bioinformatics analyses were conducted using “QIIME2” (Bolyen et al., 2018), following the official tutorials with minor modifications. Initially, raw sequence data were demultiplexed using the demux plugin, and primers were trimmed with the “cutadapt” plugin (Martin, 2011). Subsequent steps included quality filtering, denoising, merging, and removal of chimeric sequences, performed with the “DADA2” plugin (Callahan et al., 2016). Taxonomic classification of the filtered reads was carried out against the “SILVA” (release 119) V3–V4 classifier at 99% identity (Quast et al., 2012). Operational taxonomic units (OTUs) were identified as features across all samples without clustering. The resulting feature table, rooted phylogenetic tree, representative sequences, and metadata were exported from “QIIME2” for further analysis in *R* version 4.2.2 (R Core Team R, 2013).

### 2.5 Alpha and beta diversity

Alpha diversity metrics, including Chao1, Shannon, Simpson, and ACE indices based on the observed OTUs, were computed using “QIIME2.” Beta diversity was assessed via Bray–Curtis distance and Weighted UniFrac distance, utilizing the R package “VEGAN” (version 2.6-4) (Oksanen, 2015). Differences in community structure based on beta diversity were visualized using Principal Coordinate Analysis (PCoA).

### 2.6 Interconnected networks of the vaginal microbiota

Co-occurrence networks were constructed using Pearson’s correlation values greater than 0.1 and significance levels below 0.01, representing links between genera. These networks were visualized with the “qgraph” R package (version 1.9.8) (Epskamp et al., 2012).

### 2.7 Identification of microbiota or functions specific to reproductive phases

The “Seurat” package (version 4.2.3) (Stuart et al., 2019) was utilized to create Seurat objects from the relative abundance matrix of microbiotas or functional terms across samples. Reproductive phases (Group 1 to Group 5) were assigned as Idents, and data normalization was performed using the *NormalizeData* function and then scaled by *ScaleData*. The *FindAllMarkers* function, with the “test.use” parameter set to “wilcox,” was employed to identify microbiotas or biological functions specifically associated with the reproductive phases. Significance was determined at *p*-values less than 0.05.

### 2.8 Inferring pivotal microbiotas based on the random forest model

To infer pivotal microbiotas, samples were divided into training and testing sets in a 3:1 ratio. A random forest classifier was developed using the “randomForest” R package (version 4.7–1.1) (RColorBrewer and Liaw, 2018), with parameters set to “ntree = 1,000” and “nPerm = 100.” Model performance was evaluated using the testing set, with analysis and plotting of the area under the curve (AUC) performed using the “multiROC” (version 1.1.1) (Wei et al., 2018) and “ggplot2” (Wickham, 2011) (version 2_3.4.2) packages. The Mean Gini coefficient was used to assess feature importance in the classification model.

### 2.9 Prediction of microbiota functional contents

“PICRUSt” (v1.1.4) (Douglas et al., 2020) software was used to predict the functional composition of microbiota based on the OTU table. OTU data from all 16S rRNA datasets in QIIME2 were used to create BIOM files as input for PICRUSt, which predicted metagenomes from OTUs marker gene sequences using default parameters. Gene families were mapped to the “Kyoto Encyclopedia of Genes and Genomes” (KEGG) and “Clusters of Orthologous Groups of proteins” (COG) databases to obtain abundance data for KEGG and COG entries in each sample. Seurat’s *FindAllMarkers* function was used to identify significantly specific (*p* < 0.05) biological functional entries.

### 2.10 Ethics statement

The study was approved by the Ethics Committee of the PLA General Hospital (No. S2023-711-01). All methods were carried out in accordance with relevant guidelines and regulations.

### 2.11 Statistical analysis

Pearson correlation analysis was performed to evaluate cooperative or antagonistic relationships between different microbial genera. The unpaired *t*-test was applied to determine significant differences between two groups, with a significance threshold set at *p* < 0.05. The area under the curve (AUC) was used to assess the accuracy of the random forest model. Additionally, to identify differences in microbial communities between groups, *ANOVA* test were performed based on Bray–Curtis dissimilarity distance matrices. All statistical analyses and visualizations were carried out using the R programming platform (version 4.2.2).

## 3 Results

### 3.1 Investigating microbial community composition at the phylum level across reproductive phases

**Figure 1:**
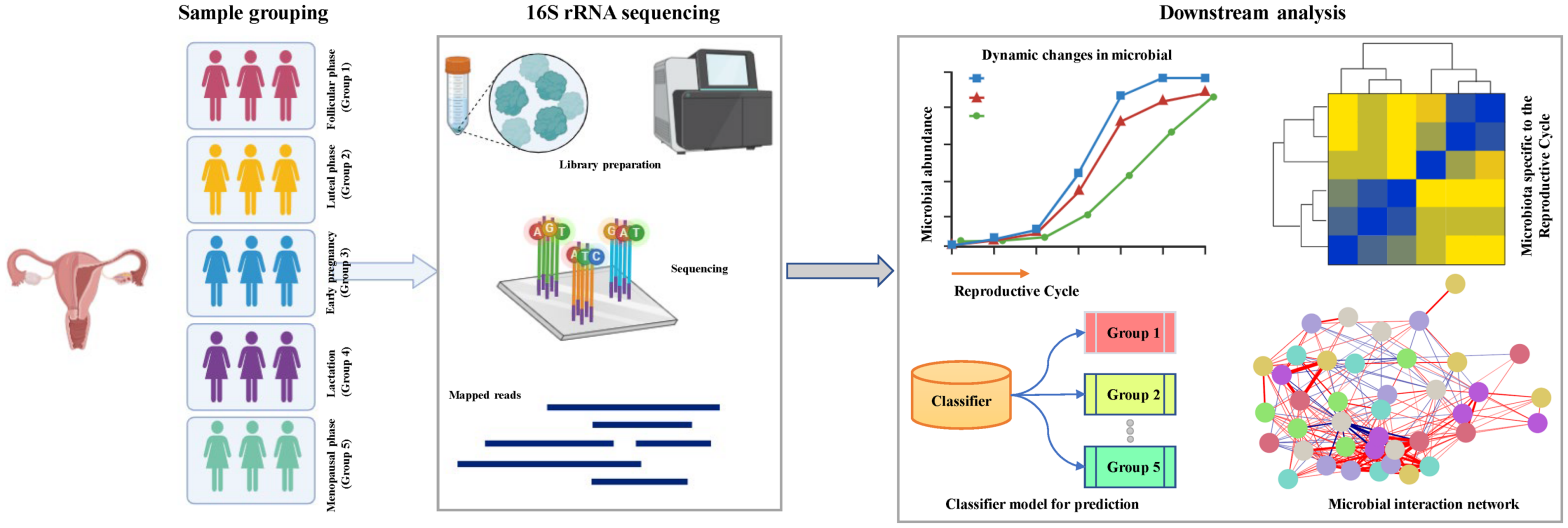
Flow chart of the study. The flowchart includes information on microbiota groups (left panel), library preparation (middle panel), 16S rRNA sequencing, and the major downstream analysis modules (right panel).

**Figure 2:**
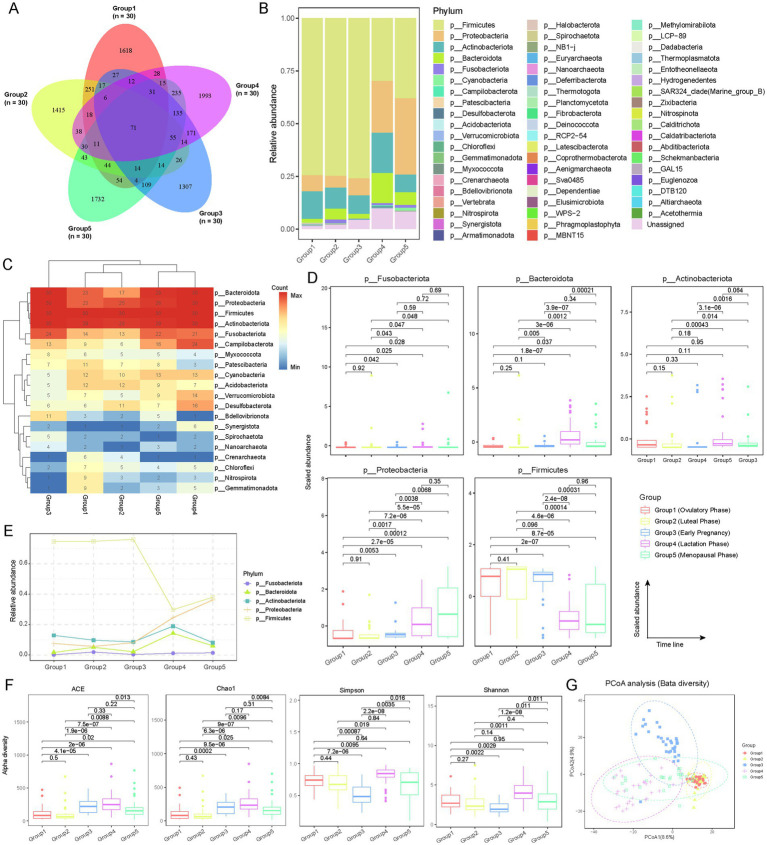
Microbial community compositions among female reproductive phases. **(A)** Venn diagram showing the overlap of operational taxonomic units (OTUs) detected at different reproductive phases. The numbers within the diagram indicate the count of detected OTUs. The labels (*n* = 30) correspond to the sample size for each group. **(B)** Relative abundance of different microbial communities (phylum level) across various groups. Different colors represent different microbial phyla. The distribution of phyla in the bar chart is arranged from top to bottom based on their average abundance in each group. **(C)** Frequency of detection of different microbial taxa across reproductive phases, indicated by sample count. Only microbial taxa detected in more than 10 samples are shown. Hierarchical clustering is applied to different groups and microbial phyla. **(D)** Box plot showing the distribution of the relative abundance of microbial taxa with an average relative abundance greater than 0.01 across different reproductive phases. Only five microbial taxa were identified. Outliers are represented by points outside the box plots. The x-axis is ordered according to the gonadal developmental timeline, with *p*-values obtained from *t*-tests. **(E)** Dynamic variations in microbial taxa with an average relative abundance greater than 0.01 among female reproductive phases. **(F)** Alpha diversity of microbial communities at different reproductive phases, assessed using ACE, Chao1, Simpson, and Shannon indices. T-tests are used to evaluate the statistical differences in alpha diversity between phases (Supplementary Table S5). **(G)** Beta diversity analysis of microbial communities at different reproductive phases using PCoA. Each point represents an individual sample, with different shapes and colors indicating the various reproductive phases.

To depict the vaginal microbiota across the female reproductive phases, we collected 150 vaginal secretion samples from five distinct phases: follicular phase (Group 1), luteal phase (Group 2), early pregnancy (Group 3), lactation (Group 4), and menopause (Group 5), with 30 samples per phase (Figure 1; Supplementary Table S1; see Materials and Methods). Utilizing 16S rRNA sequencing, we performed a comprehensive analysis of the microbial communities present in these samples. Operational Taxonomic Unit (OTU) analysis revealed distinct differences in microbial communities across the various reproductive phases (Figure 2A; Supplementary Tables S2–S4; see Materials and Methods). The number of OTUs was 2,221 in Group 1, 2067 in Group 2, 2013 in Group 3, 2,863 in Group 4, and 2,597 in Group 5. Although 71 OTUs were shared among all five phases, each phase exhibited a substantial number of unique OTUs. The proportion of phase-specific OTUs was notably high, with 72.8% (*n* = 1,618) in Group 1, 68.5% (*n* = 1,415) in Group 2, 64.9% (*n* = 1,307) in Group 3, 69.6% in Group 4, and 66.7% (*n* = 1,732) in Group 5, highlighting specific differences in the composition of microbial communities across the different reproductive phases.

To evaluate the microbial composition at the phylum level, *Firmicutes*, *Proteobacteria*, *Actinobacteriota*, *Bacteroidota*, and *Fusobacteriota* were predominant across all groups, with a cumulative average abundance exceeding 90% (Figure 2B). However, their relative abundances exhibited distinct dynamic changes. *Firmicutes* showed an average abundance exceeding 70% in Group 1, Group 2, and Group 3, but this dropped to approximately 30% in Group 4 and Group 5, indicating a declining trend with decreasing sex hormone levels. In contrast, the abundance of *Proteobacteria*, *Bacteroidota*, and *Fusobacteriota* progressively increased from Group 1 to Group 5. *Actinobacteriota* exhibited a sharp increase in Group 4, while remaining relatively stable (~10%) in the other four groups. Additionally, the abundance of unclassified (Unassigned) phyla increased from 1.4% in Group 1 to 9.8% in Group 5. We also analyzed the detection rates of the top 20 high-abundance phyla in each group. *Firmicutes*, *Proteobacteria*, *Actinobacteriota*, *Bacteroidota*, and *Fusobacteriota* had significantly higher detection rates in all samples, with *Firmicutes* detected in 100% of samples (Figure 2C). Statistical analysis confirmed significant differences in the abundance of these phyla across different phases (Figures 2D,E). *Firmicutes* were significantly (*t*-test; *p* < 0.01) more abundant in Group 1, Group 2, and Group 3 compared to Group 4 and Group 5, whereas *Proteobacteria* exhibited the opposite trend. Notably, *Bacteroidota* showed a significant increase in Group 4 (lactation) (Figures 2D,E; *t*-test; *p* < 0.01), potentially related to its roles in promoting lactose breakdown, maintaining vaginal microbiota balance, inhibiting pathogenic growth, and regulating local immune responses, which may be crucial for maternal and infant health.

To investigate microbial diversity across various reproductive phase, alpha diversity was assessed using ACE, Chao1, Simpson, and Shannon indices. The results indicated that Group 4 and Group 5 exhibited the highest diversity in most indices, while Group 1 and Group 2 generally showed lower diversity (Figure 2F; see Materials and Methods; Supplementary Table S5). Group 3 displayed moderate to high diversity levels in some indices. These differences were statistically significant, indicating distinct microbial community structures across different reproductive phases. Notably, the findings suggest a richer and more balanced microbiota during lactation and menopause. Beta diversity analysis, conducted through Principal Coordinates Analysis (PCoA), demonstrated clear differential distribution among the five groups with no apparent mixed clustering, further confirming the significant heterogeneity of microbial communities at different reproductive phases (Figure 2G; see Materials and Methods). Overall, these results reveal the dynamic characteristics of the vaginal microbiota throughout the female reproductive phase, reflecting the profound impact of differences in sex hormone levels on the composition and diversity of vaginal microbial communities.

### 3.2 Dynamic shifts and interactions in vaginal microbial genera across reproductive phases

**Figure 3:**
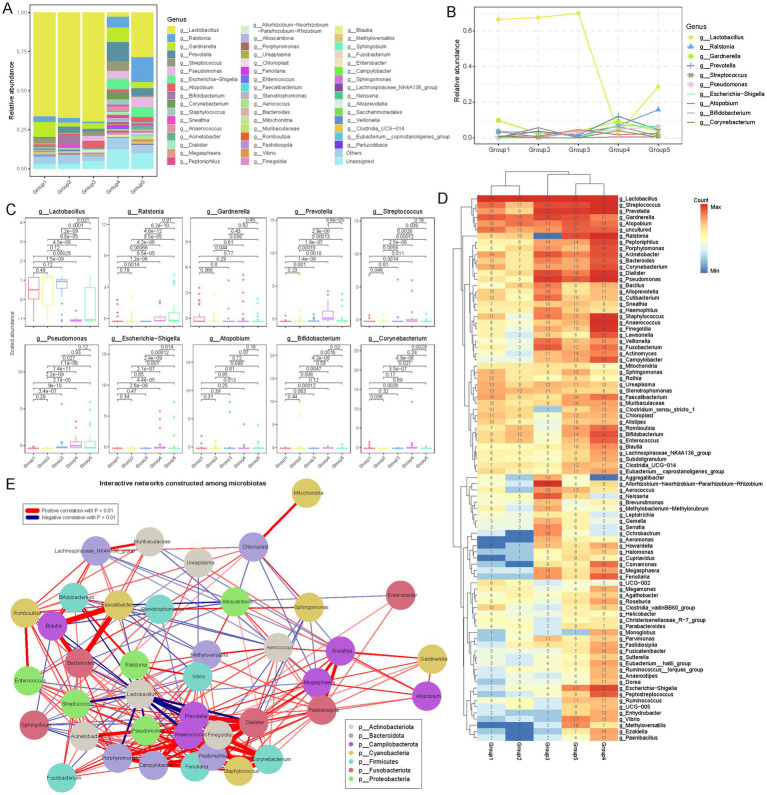
Genus-level microbial community diversity among female reproductive phases. **(A)** Stacked bar plot showing the relative abundance of different microbial communities (genus level) in each group. Different colors represent different microbial genera. In the bar chart, the distribution of genera from top to bottom is arranged in descending order of their average abundance in each group. **(B)** Dynamic variations in genera with an average relative abundance greater than 0.01 during different female reproductive phases. Interestingly, during the lactation phase, the relative composition of microbial genera shows significant differences. **(C)** Box plot showing the distribution of the abundance of microbial genera with an average relative abundance (mean abundance across all samples) greater than 0.01 in each reproductive phase (only 10 genera were identified). Points outside the box plot represent outlier samples. The horizontal axis is ordered according to the reproductive phases, and the vertical axis represents the scaled abundance. *p*-values were obtained by *t*-test. **(D)** Heatmap showing the frequency of different microbial genera detected in each reproductive phase, represented by the number of samples. Only microbial genera detected in more than 20 samples are shown. Hierarchical clustering was used to cluster different groups and microbial genera separately. **(E)** Pearson correlation coefficients were used to analyze the interactions between different microbial genera. All genera with a relative abundance greater than 0.1% were included in the network. Genera such as *Anaerococcus*, *Dialister*, *Pseudomonas*, and *Peptoniphilus* show significant synergistic effects. Interestingly, Lactobacillus shows significant antagonistic interactions with *Prevotella*, *Anaerococcus*, and *Streptococcus*, suggesting some competitive relationships among these genera in the microbial community. In the network diagram, red lines represent positive correlations, blue lines represent negative correlations, and only correlations with *p* < 0.01 are shown. Different colors in the nodes represent different phyla to which the microbial genera belong.

Through a comprehensive analysis of microbial communities at the genus level, we identified 1,074 distinct microbial genera (Supplementary Table S4). Our composition analysis highlighted *Lactobacillus*, *Ralstonia*, and *Prevotella* as the predominant genera. *Lactobacillus* exhibited substantial dominance during early reproductive phases, with relative abundances of 66.50, 67.56, and 69.70% in Group 1, Group 2, and Group 3, respectively. However, its prevalence notably declined to 28.75% in Group 4 and further to 2.69% in Group 5 (Figure 3A). This trend was consistently supported by further analyses (Figures 3B–D). Interestingly, during lactation, corresponding to the Group 4, the abundance of *Lactobacillus* experienced a significant decrease. This finding contrasts with prior studies that suggested stable or increased levels of Lactobacillus during the lactation phase (Khodayar-Pardo et al., 2014). One plausible explanation for this discrepancy is the concurrent increase in prolactin levels during lactation, which stimulates milk production, while estrogen and progesterone levels remain relatively subdued. These hormonal fluctuations likely contribute to the observed suppression of lactobacilli in the vaginal environment.

Additionally, *Ralstonia* exhibited an opposite trend to *Lactobacillus*. Its relative abundance increased gradually from 3.54% in Group 1 and 2.99% in Group 2 group to 6.96% in Group 4, eventually reaching a peak of 15.79% in Group 5 (Figures 3A,B). Frequency analysis also demonstrated a significant increase in the occurrence of *Ralstonia* in Group 4 and Group 5 (Figure 3D). This growth trend might be attributed to *Ralstonia’s* adaptability to low pH environments; as *Lactobacillus* decreases, the vaginal pH might rise, creating favorable conditions for *Ralstonia* growth. Generally, *Ralstonia* is primarily found in external environments. Lozano et al. (2021) reported its presence in the vagina, which aligns with our findings. *Prevotella* also exhibited a unique pattern of change. Its relative abundance increased from 0.64% in Group 1 group to 12.09% in Group 4, although it slightly decreased to 4.62% in Group 5, remaining significantly higher than in the earlier phases (Group 1 to Group 3; Figures 3A,B). Box plots clearly illustrated the significant increase of *Prevotella* in Group 4 (Figure 3C). This change might be related to *Prevotella’s* ability to utilize specific metabolic substrates, such as glycogen accumulation during late pregnancy and early postpartum, which could provide favorable conditions for *Prevotella* growth.

Moreover, network analysis of genus interactions revealed complex synergistic and antagonistic relationships (Figure 3E; Supplementary Table S6; see Materials and Methods). Genera such as *Anaerococcus*, *Dialister*, *Pseudomonas*, and *Peptoniphilus* exhibited significant synergistic interactions, suggesting symbiotic or functionally complementary relationships in the gonadal microenvironment. Notably, *Lactobacillus* showed clear antagonistic interactions with genera such as *Prevotella*, *Anaerococcus*, and *Streptococcus*, indicating competitive dynamics that maintain microbial balance in the gonadal ecosystem. This complex interaction network provides new insights into the ecological balance of the gonadal microbiome.

Overall, these findings underscore the nuanced genus-level composition and dynamic variations within vaginal microbial communities. Each reproductive phase, particularly lactation, presents distinct microbial patterns, while interaction analyses shed light on intricate community dynamics.

### 3.3 Specific genera associated with dynamic variations in the female reproductive phases

**Figure 4:**
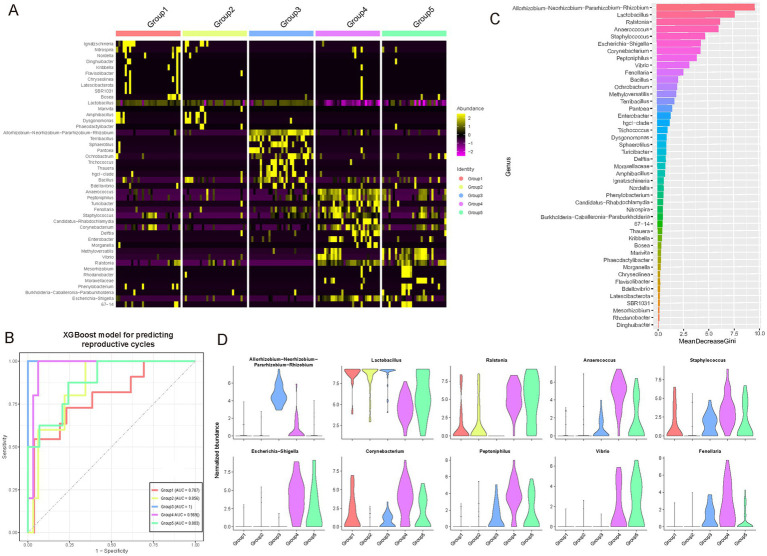
Identification of specific genera among female reproductive phases. **(A)** Heatmap showing genera specific to different reproductive phases. The color intensity in the heatmap represents the scaled relative abundance. **(B)** A multi-class classifier was constructed using a random forest model based on genera specific to different reproductive phases. The Area Under the Curve (AUC) demonstrates the classifier’s excellent performance, particularly in identifying Group 3 and Group 4, achieving an AUC of over 0.95. **(C)** Bar chart ranking the contribution of different features (microbial genera) in the random forest classifier. The x-axis represents the mean GINI coefficient, with higher values indicating a greater contribution of the corresponding genus. These key driving genera may play crucial roles during gonadal development. **(D)** Violin plots showing the dynamic variations in abundance of the top 10 key driving genera across different female reproductive phases.

To investigate genus-level differences during key phases of the female reproductive phase, we conducted specific analyses and identified microbial genera exhibiting phase-specificity (Figure 4A; see Materials and Methods; Supplementary Table S7). The heatmap illustrates the relative abundance distributions of these genera across different phases, with color intensity representing standardized relative abundance, thereby enabling the observation of abundance variation patterns. During the follicular phase (Group 1), elevated estrogen levels are associated with an increase in *Lactobacillus*, which maintains vaginal acidity and inhibits the growth of pathogenic bacteria. In the luteal phase (Group 2), increased progesterone levels correlate with a slight rise in anaerobic bacteria such as *Gardnerella vaginalis*, although lactobacilli remain predominant. Early pregnancy (Group 3) is characterized by further increases in progesterone, leading to a rise in *Lactobacillus crispatus* and other *lactobacilli*, enhancing vaginal defenses; some studies also report an increase in *Bifidobacterium*. During lactation (Group 4), the breast microbiota undergoes alterations, with increases in *Staphylococcus* and *Streptococcus*, which facilitate the establishment of the infant gut microbiota, while the vaginal microbiota gradually reverts to a non-pregnant state. In the menopausal transition (Group 5), declining estrogen levels result in a decrease in *lactobacilli* and a relative increase in anaerobic bacteria such as *Prevotella* and *Gardnerella*, along with a rise in pH levels, potentially disrupting the vaginal microbiota balance.

To delineate potential pivotal genera across distinct reproductive phases, we employed a random forest classification model built upon specific microbial taxa (Figure 4B; see Materials and Methods). Our findings underscored the model’s robust predictive capability across these phases, notably achieving an optimal area under the curve (AUC) value of 1 in Group 3. Subsequently, Group 4 demonstrated a high AUC of 0.969, whereas Group 1 exhibited the lowest at 0.787; the remaining phases surpassed 0.85. Moreover, through ranking by mean GINI coefficient, we discerned the significant contributions of various microbial genera to the model’s classification accuracy (Figure 4C; see Materials and Methods). Notably, genera such as the composite *Lactobacilli*, *Ralstonia*, *Anaerococcus*, and *Staphylococcus* prominently influenced the classification outcomes. This suggests their potential pivotal roles in reproductive gland development, where fluctuations in their abundance may closely correlate with developmental processes. Notably, *Allorhizobium-Neorhizobium-Pararhizobium-Rhizobium* (ANPR), which exhibited the highest Gini coefficient and is prevalent in Group 3 (early pregnancy), remains difficult to assess due to the absence of effective negative controls in our study, complicating the determination of its role or the potential for contamination. To further examine these genera’s dynamics, violin plots depicted the abundance distributions of the top 10 contributing genera across developmental phases (Figure 4D). These visual representations vividly illustrated each genus’s abundance characteristics, including median, quartile ranges, and outliers. For instance, *Lactobacilli* exhibited significantly heightened abundance in Group 1 compared to other phases, whereas *Ralstonia* peaked in Group 2. These dynamic patterns offer critical insights into the putative functions of these microbial genera during reproductive gland development.

In summary, these findings highlight dynamic variations in microbial communities within reproductive phases, suggesting potential for developing predictive tools based on microbial composition to assess phases of the female reproductive phase and advance women’s health management.

### 3.4 Determination of driving microbial phyla, classes, orders, families, and species in the female reproductive phases

**Figure 5:**
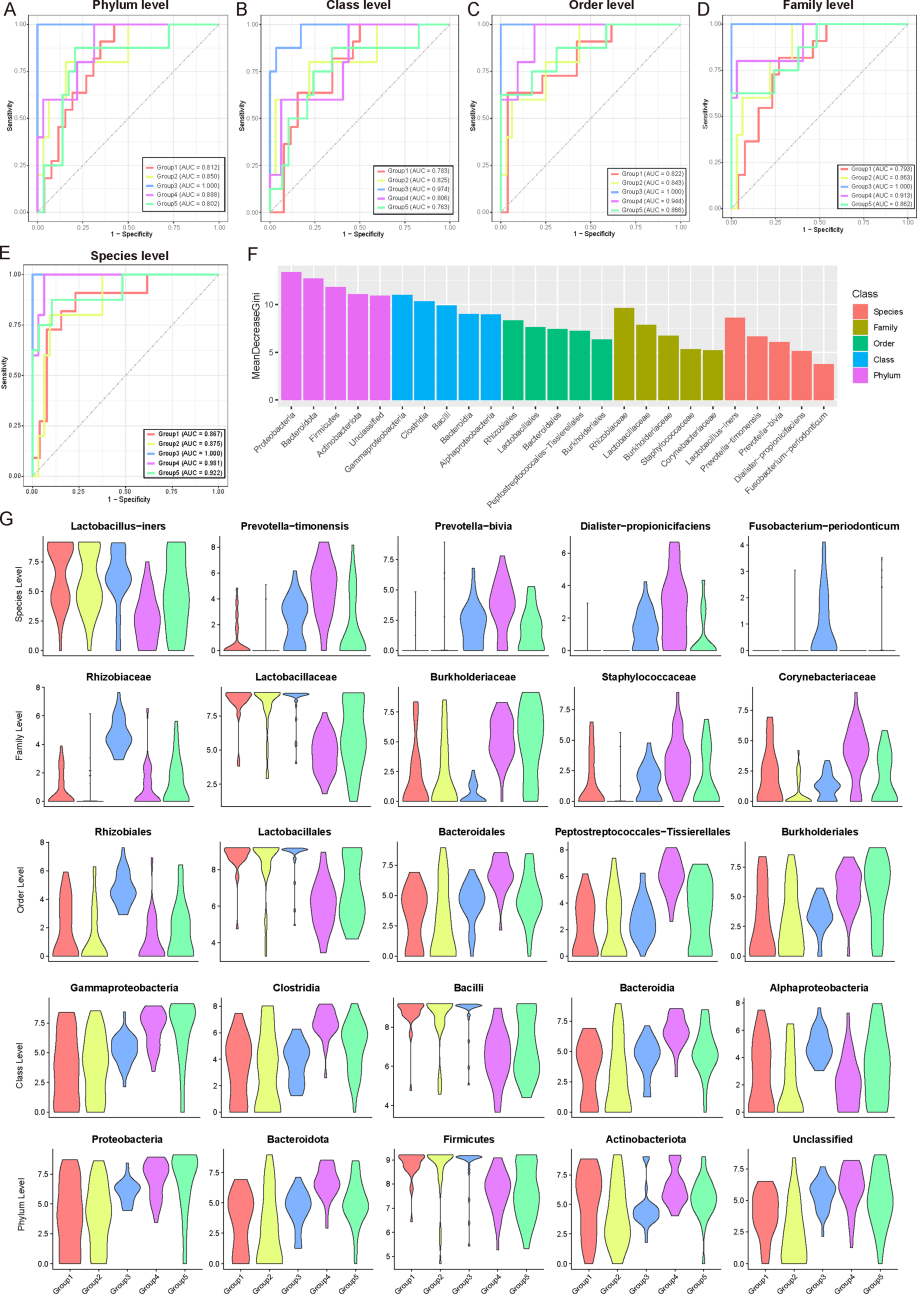
Microbial community analysis and classification across female reproductive phases. **(A–E)** Wilcoxon test was used to identify microbial communities specific to different female reproductive phases. These identified communities were then used as features to construct a multi-class classifier using a random forest model. The Area Under the Curve (AUC) indicates excellent performance of the classifier, particularly for Group 3, followed by Group 4. **(F)** Bar chart ranking the contribution of different features (microbial communities) in the random forest classifier. The x-axis represents the mean GINI coefficient, with higher values indicating a greater contribution of the corresponding feature. Different colors represent different microbial taxonomic levels. For each taxonomic level, only the top five contributing microbial communities are shown. These high-contribution features may be potential key drivers during gonadal development. **(G)** Violin plots showing the dynamic variations in abundance of the top five key driving microbial communities at different taxonomic levels across different reproductive phases.

We performed an extensive analysis of the microbiota communities integral to the female reproductive phase, examining multiple taxonomic levels from phylum to species. Utilizing the Wilcoxon test, we identified phase-specific microbial taxa at the phylum, class, order, family, and species levels. Based on these identified features, we developed random forest classifiers (Figures 5A–E; Supplementary Table S7; see Materials and Methods). These classifiers exhibited robust performance across all taxonomic levels, achieving an average AUC greater than 0.8 at the phylum level, greater than 0.78 at the class level, greater than 0.82 at the order level, greater than 0.79 at the family level, and greater than 0.86 at the species level. The species-level microbiota displayed the highest specificity throughout the female reproductive phase. The classification models demonstrated exceptional efficacy in distinguishing between Group 3 and Group 4, consistently maintaining high AUC values (Group 3, Average AUC > 0.97; Group 4, Average AUC > 0.90). This high predictive accuracy underscores the close relationship between microbial composition across various taxonomic levels and the reproductive phases.

Further analysis of feature importance within the random forest model (Figure 5F) identified key microbial taxa driving these differences at each taxonomic level. At the phylum level, *Proteobacteria*, *Bacteroidetes*, and *Firmicutes* emerged as primary contributors, reflecting their widespread ecological presence and potential roles in gonadal development. At more specific taxonomic levels, classes such as *Gammaproteobacteria*, *Clostridia*, and *Bacilli* were found to significantly contribute, likely due to their specific metabolic functions or roles in immune regulation. Notably, taxonomic groups at the order and family levels, such as *Rhizobiales*, *Lactobacillales*, *Rhizobiaceae*, and *Lactobacillaceae*, were prominently associated with microbial communities critical for maintaining microenvironmental balance and contributing essential metabolic products during reproductive phases. Similarly, species-level analyses highlighted the importance of taxa like *Lactobacillus-iners*, *Prevotella-timonensis*, and *Prevotella-bivia*, which are commonly found in reproductive tract microbiota and likely play roles in adapting to and influencing gonadal microenvironments. Figure 5G, depicted as violin plots, illustrated the abundance distributions of these influential microbial taxa across different female reproductive phases. These distributions revealed intriguing patterns, such as the higher abundance of *Lactobacillus-inners* in Group 1, gradually declining in subsequent phases, suggesting early preferences for lactobacilli in the gonadal environment (Figure 5G). Meanwhile, *Prevotella* species showed increased abundance in mid-to-late phases (Groups 3–5), indicating their potential involvement in later reproductive phases.

Overall, these integrated findings underscore the intricate dynamics of gonadal microbial communities across developmental phases. By examining microbial composition from broad taxonomic perspectives to specific species-level dynamics, these results provide a comprehensive view of how microbial community structure changes and its potential implications for gonadal health and function.

### 3.5 Predicting the metabolic functions of microbes across the female reproductive phases

**Figure 6:**
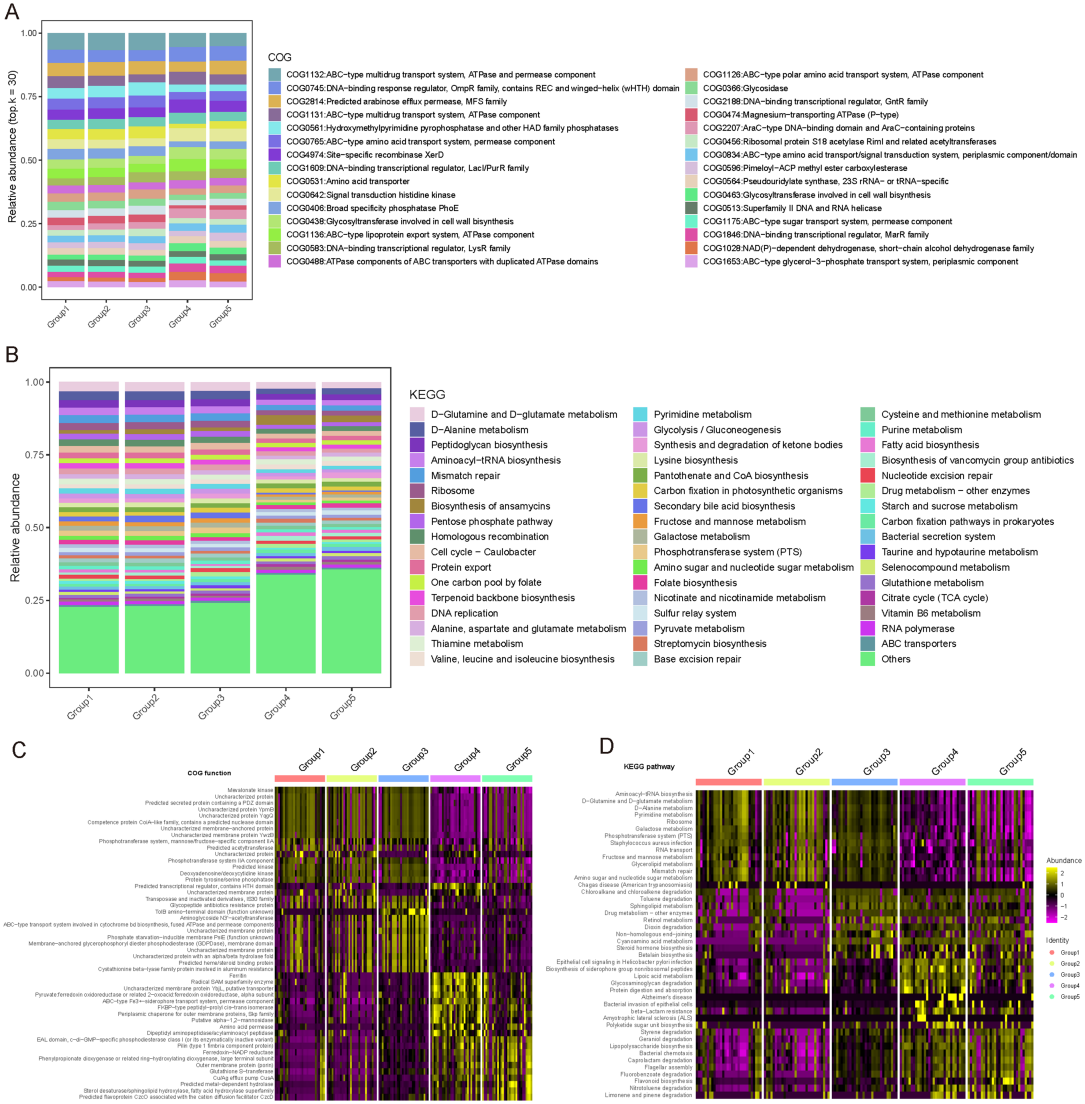
Functional profiling of COG and KEGG pathways across female reproductive phases. **(A)** Relative abundance of different Clusters of Orthologous Groups (COG) functions across gonadal groups. Different colors represent different COG entries. The bar chart displays the distribution of COG entries from top to bottom based on their average abundance in each group. Only the top 30 COG entries with the highest average relative abundance are shown; the remaining COG entries are classified as “Others.” **(B)** Relative abundance of different Kyoto Encyclopedia of Genes and Genomes (KEGG) pathways across gonadal groups. Different colors represent different KEGG entries. The bar chart displays the distribution of KEGG entries from top to bottom based on their average abundance in each group. Only the top 50 KEGG entries with the highest average relative abundance are shown; the remaining KEGG entries are classified as “Others.” **(C)** COG functions specific to different reproductive phases identified using the Wilcoxon test. The heatmap shows the relative abundance differences of these specific COG functions across different phases. **(D)** KEGG functions specific to different reproductive phases identified using the Wilcoxon test. The heatmap shows the relative abundance differences of these specific KEGG functions across different phases.

Functional composition of the vaginal microbiota across different phases of the female reproductive phase was investigated (see Materials and Methods). Our results reveal the metabolic functional characteristics of the microbiota during various phases of gonadal development and their potential physiological significance. From the COG (Clusters of Orthologous Groups) functional analysis, we observed core functions maintained at high abundance across all developmental phases, such as ABC transport systems, DNA-binding transcription regulators, signal transduction components, and glycosyltransferases involved in cell wall biosynthesis (Figure 6A). The ubiquitous presence of these functions highlights the importance of substance transport, transcription regulation, environmental sensing, and cell structure maintenance in the gonadal microbiota. KEGG pathway analysis further enriched these observations, showing high abundance of pathways related to amino acid metabolism, nucleotide metabolism, and repair across multiple phases, emphasizing their core role in the vaginal microbiota (Figure 6B).

Additionally, we noted specific functional differences in microbial adaptation to the demands of different reproductive phases, reflecting unique metabolic needs and adaptations (Figures 6C,D; Supplementary Tables S8, S9). For instance, the follicular phase (Group 1) showed enrichment in genes related to nitrotoluene degradation and flavonoid biosynthesis, which may be linked to estrogen activity regulation. The luteal phase (Group 2) was characterized by pathways involved in aromatic compound metabolism, indicating adaptation to specific metabolites. Early pregnancy (Group 3) showed enrichment in pathways associated with caprolactam and geraniol degradation, potentially involved in progesterone metabolism. During lactation (Group 4), pathways related to protein digestion, glycosaminoglycan degradation, and fatty acid metabolism were prevalent, reflecting the nutritional needs of this phase. In menopause, pathways related to steroid hormone biosynthesis and cyano-amino acid metabolism were enriched, suggesting adaptation to hormonal changes.

These findings underscore the high adaptability of the vaginal microbiota to the host’s physiological state, highlighting its potential role in maintaining ecological balance and supporting reproductive health.

## 4 Discussion

Our study explored the vaginal microbiota of 150 healthy women across different reproductive phases (follicular phase, luteal phase, early pregnancy, lactation, and menopause) using 16S rRNA gene sequencing to elucidate the dynamic characteristics of vaginal microbiota throughout the female reproductive cycle. Significant differences in the composition and diversity of vaginal microbiota were observed in tandem with the reproductive phases, underscoring the profound impact of sex hormone fluctuations on vaginal microecology.

Throughout the reproductive phases, *Firmicutes*, *Proteobacteria*, and *Actinobacteria* were the predominant components of the vaginal microbiota. However, their relative abundance exhibited substantial dynamic variations across different phases. For instance, *Firmicutes* maintain a high abundance (>70%) during the follicular phase, luteal phase, and early pregnancy but markedly decreased (approximately 30%) during lactation and menopause. This pattern aligns closely with the fluctuations in sex hormone levels, suggesting that sex hormones play a regulatory role in maintaining vaginal microecological balance. At the genus level, *Lactobacillus* dominates the early reproductive phases but significantly diminished during lactation and menopause. This finding contrasts with previous studies that indicated *Lactobacillus* might remain stable or increase during lactation (Redelinghuys et al., 2017; Yoon and Kim, 2021; Deka et al., 2021). The discrepancy may stem from variations in the ratios of sex hormones and prolactin, influenced by individual differences in hormone production, breastfeeding frequency, or other physiological factors. According to the community state type (CST) classification proposed by Ravel et al. (2011), Groups 1–3 (follicular, luteal, and early pregnancy) predominantly align with CST-I and CST-III, dominated by *Lactobacillus crispatus* and *Lactobacillus iners*, respectively. Conversely, Groups 4 (lactation) and 5 (menopause) show a pronounced shift toward CST-IV, characterized by increased microbial diversity and dominance of anaerobic genera such as *Prevotella* and *Gardnerella*. This pattern is consistent with reports that CST-IV is prevalent among healthy women of Hispanic and African American ancestry. Notably, while CST-IV and elevated microbial diversity are observed in certain healthy cohorts, increased diversity does not necessarily signify a healthier vaginal milieu. Instead, it is often accompanied by elevated vaginal pH and diminished glycogen levels factors that may facilitate pathogenic colonization and adversely impact female reproductive health (Mirmonsef et al., 2014).

One notable finding is the identification of microbial groups that predominate during specific reproductive phases. For example, the genus *Allorhizobium-Neorhizobium-Pararhizobium-Rhizobium* (ANPR) shows notable specificity during early pregnancy, with Bi et al. also reporting its presence in the vaginal microbiome (Bi et al., 2023). ANPR is typically associated with nitrogen-fixing symbiotic relationships in plant roots (Jiao et al., 2022). Its presence in the human vaginal microenvironment may reflect contamination or suggest a similar symbiotic relationship, potentially involved in metabolic processes like amino acid metabolism during early pregnancy. Given the absence of negative controls, its biological relevance should be interpreted with caution and further validated in future investigations. Functional prediction analysis revealed the metabolic adaptations of vaginal microbiota at different reproductive phases. For instance, genes related to nitrotoluene degradation and flavonoid biosynthesis, enriched during the follicular phase, might be associated with the regulation of estrogen activity. During lactation, pathways related to protein digestion, glycosaminoglycan degradation, and fatty acid metabolism are enriched, reflecting the specific nutritional needs of this phase. These functional characteristics not only reflect the adaptation of microbial communities to the host’s physiological state but also suggest their potential roles in maintaining reproductive health.

However, the research has some limitations. Firstly, the sample size is relatively small, with only 30 participants per reproductive phase, which may limit the representativeness of the findings. Additionally, all participants were recruited from Beijing, resulting in limited geographic diversity, and potentially restricting the generalizability of the results to populations with different genetic, dietary, or environmental backgrounds. Secondly, the variations in microbiota within the same individuals at different reproductive phases were not tracked, making it challenging to obtain a more comprehensive dynamic picture of vaginal microecology. Moreover, the focus was mainly on the composition and predicted functions of microbial communities without deeply exploring the mechanisms of interactions between microbes and the host. Future research could combine multi-omics data such as host gene expression, immune response, and metabolomics to comprehensively understand the complexity of the vaginal microecosystem. Longitudinal studies are also needed to monitor individual microbiome trajectories and clarify their responses to hormonal changes. Moreover, more influencing factors, such as diet, lifestyle, and environmental exposures, should be considered to explore their potential impacts on vaginal microbiota.

In summary, our study provides valuable insights into the dynamic variation of vaginal microbiota throughout the female reproductive phases. It not only reveals the close relationship between microbial communities and host physiological states but also points out directions for future research and clinical practice. With the continuous advancement of research methods and a deeper understanding of microbe-host interactions, it is anticipated that more precise and personalized strategies will be developed to optimize the management of women’s reproductive health.

## Data Availability

All raw sequencing and pre-processed data generated in this study are deposited on Zenodo [10.5281/zenodo.12819794]. Requests for access to the analysis code can be directed to the corresponding author.
