# Supplementary material for: Characterization of vaginal microbiota across female reproductive phases
Source: Front Microbiol. 2025 Jul 4;16:1599965. doi: 10.3389/fmicb.2025.1599965 (PMC12271209; doi:10.3389/fmicb.2025.1599965)
Supplement: Supplementary file 1 [file Data_Sheet_1.zip › Supplementary materials.docx]

**Supplementary materials**

**Supplementary Tables**

**Table S1.** Sample distribution across female reproductive phases.

**Table S2.** Sequencing quality metrics and read statistics for vaginal microbiome samples.

**Table S3.** Sequencing reads and OTUs statistics for vaginal microbiome samples.

**Table S4.** Relative abundance of bacterial species across the female reproductive phases.

**Table S5.** Alpha diversity metrics for vaginal microbiome samples across reproductive phases.

**Table S6.** Correlation matrix of bacterial genera in vaginal microbiome samples.

**Table S7.** Differential abundance of genera between microbial groups.

**Table S8.** Differential abundance of COG terms between microbial groups.

**Table S9.** Differential abundance of KEGG terms between microbial groups.
